# Greenberger-Horne-Zeilinger states-based blind quantum computation with entanglement concentration

**DOI:** 10.1038/s41598-017-06777-w

**Published:** 2017-09-11

**Authors:** Xiaoqian Zhang, Jian Weng, Wei Lu, Xiaochun Li, Weiqi Luo, Xiaoqing Tan

**Affiliations:** 1 Department of Computer Science, Jinan University, Guangzhou, 510632 China; 2 School of Data and Computer Science, Sun Yat-sen University, Guangzhou, 510006 China; 3 Department of Mathematics, Jinan University, Guangzhou, 510632 China

## Abstract

In blind quantum computation (BQC) protocol, the quantum computability of servers are complicated and powerful, while the clients are not. It is still a challenge for clients to delegate quantum computation to servers and keep the clients’ inputs, outputs and algorithms private. Unfortunately, quantum channel noise is unavoidable in the practical transmission. In this paper, a novel BQC protocol based on maximally entangled Greenberger-Horne-Zeilinger (GHZ) states is proposed which doesn’t need a trusted center. The protocol includes a client and two servers, where the client only needs to own quantum channels with two servers who have full-advantage quantum computers. Two servers perform entanglement concentration used to remove the noise, where the success probability can almost reach 100% in theory. But they learn nothing in the process of concentration because of the no-signaling principle, so this BQC protocol is secure and feasible.

## Introduction

Blind quantum computation (i.e. BQC)^1–7^ is still a challenging research field, where a client has not enough quantum computability, and delegates her quantum computing to the servers who have full-advanced quantum computers. In long-distance BQC, quantum entanglement plays an important role and three mainly blind entangled states have already been studied which are blind brickwork state^1^, blind topological state^2^ and Affleck-Kennedy-LiebTasaki (i.e. AKLT) state^3^. Some BQC protocols^1, 4–6^ are based on the blind brickwork state which is proposed by Broadbent *et al*.^1^. Later, Barz *et al*.^7^ demonstrated the blindness of the brickwork state. Broadbent *et al*.^1^ in 2009 proposed a single-server BQC protocol based on single-qubit states and double-server BQC protocol based on the entanglement swapping of Bell states. However, the quantum entanglement of Bell states in double-server BQC protocol^1^ will suffer quantum channel loss due to the influence of noisy channel. To solve this problem, Morimae and Fujii^4^ proposed a method of entanglement distillation to extract high-fidelity Bell states, meanwhile its security can also be guaranteed. Li *et al*.^5^ proposed a triple-server BQC protocol based on Bell states. Sheng and Zhou^6^ proposed a double-server BQC protocol based on Bell states, where the deterministic entanglement distillation can remove the noise that transforms pure entangled states into mixed entangled states. As we can see that the aims of BQC protocols^1, 4–6^ are all to obtain the single-qubit states $|{\pm }_{{\theta }_{i}}\rangle$ with ${\theta }_{i}\in \{0,\frac{\pi }{4},\frac{2\pi }{4},\ldots ,\frac{7\pi }{4}\}$ to create the blind brickwork states^1^. The other two blind graph states^2, 3^ can also be used to perform BQC successfully. The Raussendorf-Harrington-Goyal (i.e. RHG) lattice^2^, which the blindness is guaranteed in a topological manner, is used to perform four quantum measurements {*X*, *Y*, *Z*, *T*} only known by clients. Compared with the cluster states, AKLT states can be prepared efficiently and simply in linear optics with biphotons^8^. Recently, more and more interesting BQC protocols are proposed^9–18^. In BQC, the quantum channel noise is still an urgent problem. Previous works^4, 6, 14^ have studied quantum channel noises in BQC protocols. For example, Takeuchi *et al*.^14^ proposed three BQC protocols based on decoherence-free subspace (i.e. DFS) to resist the collective noise of quantum channel.

The new BQC protocol is based on maximally GHZ entangled states, where there are three participants (a client Alice, two servers Bob and Charlie). The BQC protocol is divided into four steps. First, Bob prepares initial GHZ states, remains one photon and sends other two photons to Alice. Alice disturbs the orders of two photons and sends to Charlie. Second, Bob and Charlie perform entanglement concentration to get ideal maximally entangled states, where two identical less-entangled states can be used to concentrate a maximally entangled state by two-step parity check and project measurements. Third, Bob performs Pauli operations on his photons under Alice’s instruction. Then Charlie performs measurement on one photon with the basis {|0〉, |1〉}. Alice randomly chooses *θ*_*i*_ $(\in \,\{\mathrm{0,}\frac{\pi }{4},\ldots ,\frac{7\pi }{4}\})$ and sends to Charlie. Charlie performs measurement on the other photon and Bob gets the single-qubit. Finally, Alice and Bob perform single-server BQC protocol.

This BQC protocol has four contributions. First, two servers can communication with each other without degrading the security. Second, it does not need a trusted center. The task of preparing initial entangled states can be assigned to Bob. Third, Bob and Charlie don’t need to exchange their classical information. If they collude, they don’t know any information about Alice’s inputs, outputs and algorithms. The last one, entanglement concentration can be used to remove the channel noise.

## Results

### BQC protocol based on maximally GHZ entangled states

**Figure 1:**
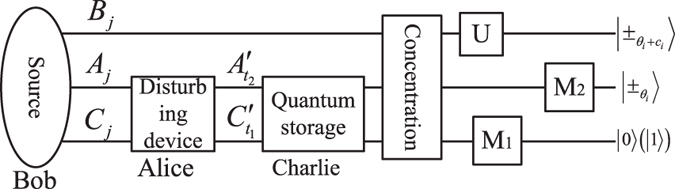
Schematic diagram of BQC protocol is based on maximally GHZ entangled states with *z*-basis ({|0〉, |1〉}) measurement *M*_1_, basis $|0\rangle \pm {e}^{-i{\theta }_{i}}|1\rangle$ measurement *M*_2_, Pauli operations *U*, Bell measurement BM, Charlie’s measurement outcome *c*_*i*_. The order of photons sequences *A*_*j*_ and *C*_*j*_ ($j\in \{1,2,3,\ldots ,n\}$) are disturbed and rewritten as ${A}_{{t}_{2}}^{\prime }$ and ${C}_{{t}_{1}}^{\prime }$ (*t*_1_, ${t}_{2}\in \{1,2,3,\ldots ,n\}$). Photons ${A}_{{t}_{2}}^{\prime }$ and ${C}_{{t}_{1}}^{\prime }$ belong to Charlie, and *B*_*j*_ belongs to Bob, where ${A}_{{t}_{2}}^{\prime }$, *B*_*j*_ and ${C}_{{t}_{1}}^{\prime }$ (*t*_1_ ≠ *t*_2_ ≠ *j*) belong to a GHZ state.

Photons are the best physical systems for the long-distance transmission of entangled states, thus entangled photons states are used as quantum information carriers in BQC. In this BQC protocol, we use |0〉 and |1〉 to express photons. In entanglement concentration, we use |*H*〉 and |*V*〉 to express photons, where |*H*〉 is equal to |0〉 and |*V*〉 is equal to |1〉. In this section, we propose the BQC protocol based on maximally GHZ photons entangled states ${|GHZ\rangle }_{{A}_{j}{B}_{j}{C}_{j}}=\frac{1}{2}(|001\rangle +|010\rangle +|100\rangle +|111\rangle )$ ($j=1,2,\ldots ,n$) (Fig. 1). The cross-Kerr nonlinear can be used to construct a CNOT gate in ref. 19. There are also many other methods to realize it^19–23^. In the BQC protocol, we suppose that these quantum devices are all ideal. The client owns quantum channels with two servers and quantum disturbing device.

- Bob generates enough maximally GHZ entangled states ${|GHZ\rangle }_{{A}_{j}{B}_{j}{C}_{j}}$, where the subscripts *A*_*j*_, *B*_*j*_ and *C*_*j*_ represents photons *A*_*j*_, *B*_*j*_ and *C*_*j*_. Bob keeps photons sequences *S*_*B*_ = [*B*_1_, *B*_2_, …, *B*_*n*_] and sends photons sequences *S*_*A*_ = [*A*_1_, *A*_2_, …, *A*_*n*_] and *S*_*C*_ = [*C*_1_, *C*_2_, …, *C*_*n*_] to Alice successively. After receiving photons sequences, Alice disturbs the order of photons sequences *S*_*A*_ and *S*_*C*_. The reordered photons sequences are rewritten as *S*_*A*′_ = [${A}_{1}^{\prime}$, ${A}_{2}^{\prime}$, …, ${A}_{n}^{\prime}$] and *S*_*C*′_ = [${C}_{1}^{\prime}$, ${C}_{2}^{\prime}$, …, ${C}_{n}^{\prime}$], meanwhile ${|GHZ\rangle }_{{A}_{j}{B}_{j}{C}_{j}}$ is remarked as ${|GHZ\rangle }_{{A}_{{t}_{2}}^{{\prime}}{B}_{j}{C}_{{t}_{1}}^{{\prime}}}({t}_{1},{t}_{2}\in \{1,2,\cdots ,n\})$. The orders of photons sequences *S*_*A*′_ and *S*_*C*′_ are different and only known by Alice. Then Alice sends photons sequences *S*_*A*′_ and *S*_*C*′_ to Charlie. Due to the effect of quantum channel noise, the maximally entangled states ${|GHZ\rangle }_{{A}_{{t}_{2}}^{\prime }{B}_{j}{C}_{{t}_{1}}^{\prime }}$ evolve into less-entangled states ${|GHZ^{\prime} \rangle }_{{A}_{{t}_{2}}^{\prime }{B}_{j}{C}_{{t}_{1}}^{\prime}}=\alpha |001\rangle +\beta |010\rangle +\delta |100\rangle +\eta |111\rangle$, where |*α*|^2^ + |*β*|^2^ + |*δ*|^2^ + |*η*|^2^ = 1. In order to get states ${|GHZ\rangle }_{{A}_{{t}_{2}}^{\prime }{B}_{j}{C}_{{t}_{1}}^{\prime }}$, Bob and Charlie firstly perform entanglement concentration.
- Bob performs one of four operations {*I*, *σ*_*x*_, *iσ*_*y*_, *σ*_*z*_} randomly chosen by Alice on photons *B*_*j*_ and ${|GHZ\rangle }_{{A}_{{t}_{2}}^{\prime }{B}_{j}{C}_{{t}_{1}}^{\prime }}$ states evolve into one of four states $\{{|GH{Z}_{1}\rangle }_{{A}_{{t}_{2}}^{\prime }{B}_{j}{C}_{{t}_{1}}^{\prime }}$, ${|GH{Z}_{2}\rangle }_{{A}_{{t}_{2}}^{\prime }{B}_{j}{C}_{{t}_{1}}^{\prime }}$, ${|GH{Z}_{3}\rangle }_{{A}_{{t}_{2}}^{\prime }{B}_{j}{C}_{{t}_{1}}^{\prime }}$, ${|GH{Z}_{4}\rangle }_{{A}_{{t}_{2}}^{\prime }{B}_{j}{C}_{{t}_{1}}^{\prime }}\}$.

1 $$\begin{array}{rcl}\mathop{\to }\limits^{I}{|GH{Z}_{1}\rangle }_{{A}_{{t}_{2}}^{\prime }{B}_{j}{C}_{{t}_{1}}^{\prime }} & = & \frac{1}{2}(|001\rangle +|010\rangle +|100\rangle +|111\rangle )\\  & = & \frac{1}{2\sqrt{2}}({|{\psi }^{+}\rangle }_{{A}_{{t}_{2}}^{\prime }{B}_{j}}{|0\rangle }_{{C}_{{t}_{1}}^{\prime }}+{|{\varphi }^{+}\rangle }_{{A}_{{t}_{2}}^{\prime }{B}_{j}}{|1\rangle }_{{C}_{{t}_{1}}^{\prime }}),\\ \mathop{\to }\limits^{{\sigma }_{x}}{|GH{Z}_{2}\rangle }_{{A}_{{t}_{2}}^{\prime }{B}_{j}{C}_{{t}_{1}}^{\prime }} & = & \frac{1}{2}(|011\rangle +|000\rangle +|110\rangle +|101\rangle )\\  & = & \frac{1}{2\sqrt{2}}({|{\varphi }^{+}\rangle }_{{A}_{{t}_{2}}^{\prime }{B}_{j}}{|0\rangle }_{{C}_{{t}_{1}}^{\prime }}+{|{\psi }^{+}\rangle }_{{A}_{{t}_{2}}^{\prime }{B}_{j}}{|1\rangle }_{{C}_{{t}_{1}}^{\prime }}),\\ \mathop{\to }\limits^{i{\sigma }_{y}}{|GH{Z}_{3}\rangle }_{{A}_{{t}_{2}}^{\prime }{B}_{j}{C}_{{t}_{1}}^{\prime }} & = & \frac{1}{2}(-\,|011\rangle +|000\rangle -|110\rangle +|101\rangle )\\  & = & \frac{1}{2\sqrt{2}}({|{\varphi }^{-}\rangle }_{{A}_{{t}_{2}}^{\prime }{B}_{j}}{|0\rangle }_{{C}_{{t}_{1}}^{\prime }}-{|{\psi }^{-}\rangle }_{{A}_{{t}_{2}}^{\prime }{B}_{j}}{|1\rangle }_{{C}_{{t}_{1}}^{\prime }}),\\ \mathop{\to }\limits^{{\sigma }_{z}}{|GH{Z}_{4}\rangle }_{{A}_{{t}_{2}}^{\prime }{B}_{j}{C}_{{t}_{1}}^{\prime }} & = & \frac{1}{2}(|001\rangle -|010\rangle +|100\rangle -|111\rangle )\\  & = & \frac{1}{2\sqrt{2}}(-\,{|{\psi }^{-}\rangle }_{{A}_{{t}_{2}}^{\prime }{B}_{j}}{|0\rangle }_{{C}_{{t}_{1}}^{\prime }}+{|{\varphi }^{-}\rangle }_{{A}_{{t}_{2}}^{\prime }{B}_{j}}{|1\rangle }_{{C}_{{t}_{1}}^{\prime }}).\end{array}$$

Since the orders of sequences *S*_*A*′_, *S*_*B*_ and *S*_*C*′_ are different, both Bob and Charlie cannot know which state ${|GH{Z}_{u}\rangle }_{{A}_{{t}_{2}}^{\prime }{B}_{j}{C}_{{t}_{1}}^{\prime }}$ (*u*∈{1, 2, 3, 4}) they shared.

- Charlie performs measurement on photons ${C}_{{t}_{1}}^{\prime }$ using the basis {|0〉, |1〉} under the guidance of Alice. Alice randomly chooses ${\theta }_{i}\in \{0,\pi \mathrm{/4},2\pi \mathrm{/4},\ldots ,7\pi \mathrm{/4}\}$ and sends to Charlie. Charlie performs measurement on the basis $\{|0\rangle \pm {e}^{-i{\theta }_{i}}|1\rangle \}$ and Bob obtains photons states $|{\pm }_{{\theta }_{i}+{c}_{i}\pi }\rangle$, where *c*_*i*_ (∈{0, 1}) is Charlie’s measurement outcome. Because the orders of ${A}_{{t}_{2}}^{\prime }$ and *B*_*j*_ are different, Bob can not know anything even if Charlie tells the value of *θ*_*i*_ to Bob.
- Alice, Bob and Charlie repeat (1–3) steps such that Bob obtains single-photon states $\underset{i=1}{\overset{n}{\bigotimes }}|{\pm }_{{\theta }_{i}+{c}_{i}\pi }\rangle$ successfully. The remaining steps are the same as steps (2–3) of the BFK protocol^1^ or steps (2–5) of blind topological BQC protocol^2^. The blindness of graph states and the correctness of quantum computation have already been exhibited in refs 1 and 2 in detail.

In the step 1 of this BQC, entanglement concentration is used to remove the noise. In the following, the process of entanglement concentration is showed with optical system.

### Entanglement concentration of pure maximally GHZ entangled state

In a practical transmission, there exist two kinds of quantum channel noises, i.e. pure maximally entangled states evolve into mixed states or less-entangled states. Entanglement purification^24–28^ is applied to extract high-fidelity maximally entangled states from mixed entangled states. Entanglement concentration^29–45^ is often used to distill less-entangled states into pure maximally entangled states by local operations and classical communication (i.e. LOCC). Bennett *et al*.^29^ firstly proposed an entanglement concentration protocol by using Schmidt projection. In 2003, Zhao *et al*.^42^ not only demonstrated the entanglement concentration scheme in ref. 30 but also verified a quantum repeater in experiment. Li *et al*.^39^ proposed two protocols to concentrate hyper-entangled GHZ states by using a single-photon state of two freedoms and two less-entangled states respectively. Sheng *et al*.^32^ proposed to concentrate arbitrary W states by using two steps. Afterwards, a universal concentration scheme of an arbitrary less-entangled N-photon W state is proposed in ref. 43. Here, we consider a special quantum channel noise, i.e. pure maximally entangled states evolve into less-entangled states, which can be distilled by entanglement concentration. In the following, we give the entanglement concentration of GHZ states that were experimentally prepared in refs 46–48.

#### The first round of entanglement concentration

In the BQC, the maximally GHZ states can be rewritten in the form of

2 $${|GHZ\rangle }_{{a}_{1}{b}_{1}{c}_{1}}=\frac{1}{2}(|HHV\rangle +|HVH\rangle +|VHH\rangle +|VVV\rangle ),$$

where we define |*H*〉 = |0〉 and |*V*〉 = |1〉. The subscripts *a*_1_, *b*_1_ and *c*_1_ represent the spatial-mode of photons ${A}_{{t}_{2}}^{\prime }$, *B*_*j*_ and ${C}_{{t}_{1}}^{\prime }$. We consider the noisy model that pure maximally entangled states evolve pure less entangled states. Suppose less-entangled pure photons states are

3 $${|GHZ^{\prime} \rangle }_{{a}_{1}{b}_{1}{c}_{1}}=\alpha |HHV\rangle +\beta |HVH\rangle +\delta |VHH\rangle +\eta |VVV\rangle ,$$

where four real numbers *α*, *β*, *δ*, *η* satisfy |*α*|^2^ + |*β*|^2^ + |*δ*|^2^ + |*η*|^2^ = 1.

**Figure 2:**
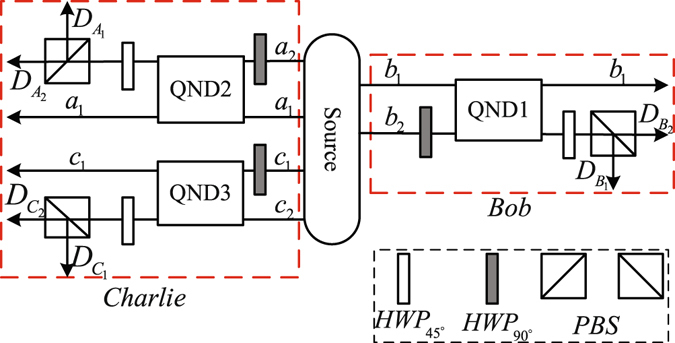
The schematic diagram of polarization-entanglement concentration. The sources is used to produce polarization-entangled states. Photons *a*_1_(*a*_2_) and *c*_1_(*c*_2_) belong to Charlie, where Bob retains photons *b*_1_(*b*_2_). HWP is half-wave plate which *HWP*_90°_ flips the horizontal and vertical polarization states. *HWP*_45°_ just likes a Hadamard operation to rotate horizontal and vertical polarization states. The polarizing beam splitters (PBSs) are used to transmit horizontal polarization |*H*〉 and reflect vertical polarization |*V*〉. *QND*_*i*_ (with *i* = 1, 2, 3) represents quantum nondemolition detections. Detectors ${D}_{{B}_{1}}$ and ${D}_{{B}_{2}}$ belong to Bob, ${D}_{{A}_{1}}$, ${D}_{{A}_{2}}$, ${D}_{{C}_{1}}$ and ${D}_{{C}_{2}}$ belong to Charlie.

Two identical less-entangled states, which the parameters are all unknown, can distill a maximally entangled state in Eq. (2). The schematic of entanglement concentration is shown in Fig. 2. Here, only Alice knows whether entanglement concentration is successful and the correct orders of ${A}_{{t}_{2}}^{{\prime }}$, *B*_*j*_ and ${C}_{{t}_{1}}^{{{\rm{\prime }}}}$.

After passing *HWP*_90°_, the state ${|GHZ^{\prime} \rangle }_{{a}_{1}{b}_{1}{c}_{1}}$ evolves to

4 $${|GHZ^{\prime} \rangle }_{{a}_{2}{b}_{2}{c}_{2}}=\alpha |VVH\rangle +\beta |VHV\rangle +\delta |HVV\rangle +\eta |HHH\rangle ,$$

where polarization photons *a*_1_, *b*_1_ and *c*_1_ are flipped and relabeled as *a*_2_, *b*_2_ and *c*_2_.

The entanglement concentration is divided into two steps. In the first step, the system composed of six photons is

5 $$\begin{array}{rcl}{|{\rm{\Psi }}\rangle }_{{a}_{1}{b}_{1}{c}_{1}{a}_{2}{b}_{2}{c}_{2}} & = & {|GHZ^{\prime} \rangle }_{{a}_{1}{b}_{1}{c}_{1}}\otimes {|GHZ^{\prime} \rangle }_{{a}_{2}{b}_{2}{c}_{2}}\\  & = & [{\alpha }^{2}|HHVVVH\rangle +{\beta }^{2}|HVHVHV\rangle \\  &  & +{\delta }^{2}|VHHHVV\rangle +{\eta }^{2}|VVVHHH\rangle ]\\  &  & +[\alpha \beta (|HHVVHV\rangle +|HVHVVH\rangle )\\  &  & +\delta \eta (|HHVHVV\rangle +|VHHVVH\rangle )]\\  &  & +[\alpha \delta (|HVHHHH\rangle +|VVVVHV\rangle )\\  &  & +\beta \eta (|VHHHHH\rangle +|VVVHVV\rangle )]\\  &  & +[\alpha \eta |HHVHHH\rangle +|VVVVVH\rangle \\  &  & +\beta \delta (|HVHHVV\rangle +|VHHVHV\rangle )]\end{array}$$

**Figure 3:**
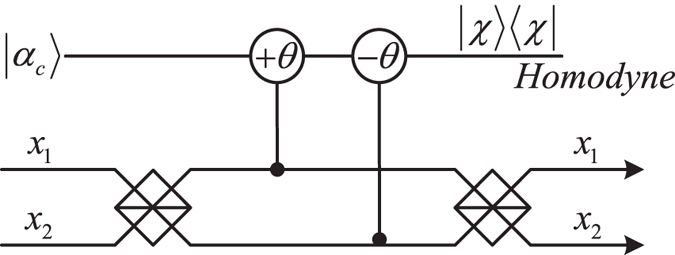
Schematic diagram of QND^49^. ±*θ* = *χt* represents the cross-Kerr nonlinearity media that introduces the phase shift *θ* when photons pass through the media. |*χ*〉 〈*χ*| is homodyne measurement that can distinguish different phase shifts. The signal photons |*α*_1_〉, |*α*_2_〉 and |*α*_3_〉 are related to *a*_1_ and *a*_2_, *b*_1_ and *b*_2_, *c*_1_ and *c*_2_ respectively. Here *x*_1_ and *x*_2_ can be specifically expressed as *a*_1_ and *a*_2_ (*b*_1_ and *b*_2_, *c*_1_ and *c*_2_).

After both *a*_1_ and *a*_2_ (*b*_1_ and *b*_2_, *c*_1_ and *c*_2_) pass parity check device (Fig. 3), Bob and Charlie can get some specific quantum state by choosing phase shifts. Here, we suppose that Bob and Charlie are honest to perform the entanglement concentration. The concrete process of the parity check device is given in Methods.

For *b*_1_ and *b*_2_, *a*_1_ and *a*_2_, *c*_1_ and *c*_2_, if Bob and Charlie all choose ±2*θ* phase shifts of odd-parity check states, the state is

6 $$\begin{array}{rcl}{|{\phi }_{1}\rangle }_{{a}_{1}{b}_{1}{c}_{1}{a}_{2}{b}_{2}{c}_{2}} & = & {\alpha }^{2}|HHV\rangle |VVH\rangle +{\beta }^{2}|HVH\rangle |VHV\rangle \\  &  & +{\delta }^{2}|VHH\rangle |HVV\rangle +{\eta }^{2}|VVV\rangle |HHH\rangle \end{array}$$

with the probability ${p}_{11}^{1}={\alpha }^{4}+{\beta }^{4}+{\delta }^{4}+{\eta }^{4}$, where ${p}_{vj}^{m}$ represents the probability of obtaining ${|{\phi }_{1}\rangle }_{{a}_{1}{b}_{1}{c}_{1}{a}_{2}{b}_{2}{c}_{2}}$ with the number of rounds *v* $(v=1,2,3,\ldots ,k)$, the number of steps *j* (*j* = 1, 2) in *v*th round and the quantum state *m* (*m* = 1, 2, 3, 4) in *j*th step of *v*th round.

If Bob chooses 0 phase shift of even-parity check states for *b*_1_ and *b*_2_, Charlie chooses 0 phase shift of even-parity check states for *c*_1_ and *c*_2_, and ±2*θ* phase shift of odd-parity check states for *a*_1_ and *a*_2_, the state is

7 $$\begin{array}{rcl}{|{\phi }_{2}\rangle }_{{a}_{1}{b}_{1}{c}_{1}{a}_{2}{b}_{2}{c}_{2}} & = & \alpha \beta (|HHV\rangle |VHV\rangle +|HVH\rangle |VVH\rangle )\\  &  & +\delta \eta (|VHH\rangle |HHH\rangle +|VVV\rangle |HVV\rangle )\end{array}$$

with the probability ${p}_{11}^{2}=\mathrm{2(}{\alpha }^{2}{\beta }^{2}+{\delta }^{2}{\eta }^{2})$.

If Bob chooses ±2*θ* phase shift of odd-parity check states for *b*_1_ and *b*_2_, Charlie chooses 0 phase shifts of even-parity check states for *a*_1_ and *a*_2_, *c*_1_ and *c*_2_, the state is

8 $$\begin{array}{rcl}{|{\phi }_{3}\rangle }_{{a}_{1}{b}_{1}{c}_{1}{a}_{2}{b}_{2}{c}_{2}} & = & \alpha \delta (|HHV\rangle |HVV\rangle +|VHH\rangle |VVH\rangle )\\  &  & +\beta \eta (|HVH\rangle |HHH\rangle +|VVV\rangle |VHV\rangle )\end{array}$$

with the probability ${p}_{11}^{3}=\mathrm{2(}{\alpha }^{2}{\delta }^{2}+{\beta }^{2}{\eta }^{2})$.

If Bob chooses 0 phase shift of even-parity check state for *b*_1_ and *b*_2_, Charlie chooses ±2*θ* phase shift of odd-parity check states for *c*_1_ and *c*_2_, and 0 phase shift of even-parity check states for *a*_1_ and *a*_2_, the state is

9 $$\begin{array}{rcl}{|{\phi }_{4}\rangle }_{{a}_{1}{b}_{1}{c}_{1}{a}_{2}{b}_{2}{c}_{2}} & = & \alpha \eta (|HHV\rangle |HHH\rangle +|VVV\rangle |VVH\rangle )\\  &  & +\beta \delta (|HVH\rangle |HVV\rangle +|VHH\rangle |VHV\rangle )\end{array}$$

with the probability ${p}_{11}^{4}=\mathrm{2(}{\alpha }^{2}{\eta }^{2}+{\beta }^{2}{\delta }^{2})$.

We give an example for PBSs measurement. After passing through *HWP*_45°_, ${|{\phi }_{1}\rangle }_{{a}_{1}{b}_{1}{c}_{1}{a}_{2}{b}_{2}{c}_{2}}$ evolves into

10 $$\begin{array}{ll}\to  & {({\alpha }^{2}|HHV\rangle +{\beta }^{2}|HVH\rangle +{\delta }^{2}|VHH\rangle +{\eta }^{2}|VVV\rangle )}_{{a}_{1}{b}_{1}{c}_{1}}\\  & \times {(|HHH\rangle +|VVV\rangle )}_{{a}_{2}{b}_{2}{c}_{2}}\\  & +{({\alpha }^{2}|HHV\rangle -{\beta }^{2}|HVH\rangle -{\delta }^{2}|VHH\rangle +{\eta }^{2}|VVV\rangle )}_{{a}_{1}{b}_{1}{c}_{1}}\\  & \times {(|HHV\rangle +|VVH\rangle )}_{{a}_{2}{b}_{2}{c}_{2}}\\  & +{(-{\alpha }^{2}|HHV\rangle +{\beta }^{2}|HVH\rangle -{\delta }^{2}|VHH\rangle +{\eta }^{2}|VVV\rangle )}_{{a}_{1}{b}_{1}{c}_{1}}\\  & \times {(|HVH\rangle +|VHV\rangle )}_{{a}_{2}{b}_{2}{c}_{2}}\\  & +{(-{\alpha }^{2}|HHV\rangle -{\beta }^{2}|HVH\rangle +{\delta }^{2}|VHH\rangle +{\eta }^{2}|VVV\rangle )}_{{a}_{1}{b}_{1}{c}_{1}}\\  & \times {(|HVV\rangle +|VHH\rangle )}_{{a}_{2}{b}_{2}{c}_{2}}.\end{array}$$

If the detectors ${D}_{{A}_{1}}$, ${D}_{{B}_{1}}$, ${D}_{{C}_{1}}$ (or ${D}_{{A}_{2}}$, ${D}_{{B}_{2}}$, ${D}_{{C}_{2}}$) are triggered, we will get

11 $${|{\phi }_{11}^{\mathrm{(1)}}\rangle }_{{a}_{1}{b}_{1}{c}_{1}}^{1}={\alpha }^{2}|HHV\rangle +{\beta }^{2}|HVH\rangle +{\delta }^{2}|VHH\rangle +{\eta }^{2}|VVV\rangle ,$$

where ${|{\phi }_{vj}^{(\gamma )}\rangle }_{{a}_{1}{b}_{1}{c}_{1}}^{m}$ represents the quantum state with the number of rounds *v* $(v=1,2,3,\ldots ,k)$, the number of steps *j* (*j* = 1, 2) in *v*th round, the quantum state *m* (*m* = 1, 2, 3, 4) in *j*th step of *v*th round, and the quantum state (*γ*) (*γ* = 1, 2, 3, 4) of PBSs measurement for the states ${|{\phi }_{\varepsilon }\rangle }_{{a}_{1}{b}_{1}{c}_{1}{a}_{2}{b}_{2}{c}_{2}}$ (*ε* = 1, 2, 3, 4).

If the detectors ${D}_{{A}_{1}}$, ${D}_{{B}_{1}}$, ${D}_{{C}_{2}}$ (or ${D}_{{A}_{2}}$, ${D}_{{B}_{2}}$, ${D}_{{C}_{1}}$) are triggered, we get

12 $${|{\phi }_{11}^{\mathrm{(2)}}\rangle }_{{a}_{1}{b}_{1}{c}_{1}}^{1}={\alpha }^{2}|HHV\rangle -{\beta }^{2}|HVH\rangle -{\delta }^{2}|VHH\rangle +{\eta }^{2}|VVV\rangle .$$

Bob and Charlie perform unitary transformation ${\sigma }_{z}^{B}\otimes {\sigma }_{z}^{A}$ on photons *a*_1_ and *b*_1_ of state ${|{\phi }_{11}^{\mathrm{(2)}}\rangle }_{{a}_{1}{b}_{1}{c}_{1}}^{1}$ to get ${|{\phi }_{11}^{\mathrm{(1)}}\rangle }_{{a}_{1}{b}_{1}{c}_{1}}^{1}$.

If the detectors ${D}_{{A}_{1}}$, ${D}_{{B}_{2}}$, ${D}_{{C}_{1}}$ (or ${D}_{{A}_{2}}$, ${D}_{{B}_{1}}$, ${D}_{{C}_{2}}$) are triggered, we will get

13 $${|{\phi }_{11}^{\mathrm{(3)}}\rangle }_{{a}_{1}{b}_{1}{c}_{1}}^{1}=-{\alpha }^{2}|HHV\rangle +{\beta }^{2}|HVH\rangle -{\delta }^{2}|VHH\rangle +{\eta }^{2}|VVV\rangle .$$

Charlie performs unitary transformation ${\sigma }_{z}^{A}\otimes {\sigma }_{z}^{C}$ on photons *a*_1_ and *c*_1_ of state ${|{\phi }_{11}^{\mathrm{(3)}}\rangle }_{{a}_{1}{b}_{1}{c}_{1}}^{1}$ to get ${|{\phi }_{11}^{\mathrm{(1)}}\rangle }_{{a}_{1}{b}_{1}{c}_{1}}^{1}$.

If the detectors ${D}_{{A}_{1}}$, ${D}_{{B}_{2}}$, ${D}_{{C}_{2}}$ (or ${D}_{{A}_{2}}$, ${D}_{{B}_{1}}$, ${D}_{{C}_{1}}$) are triggered, we will get

14 $${|{\phi }_{11}^{\mathrm{(4)}}\rangle }_{{a}_{1}{b}_{1}{c}_{1}}^{1}=-{\alpha }^{2}|HHV\rangle -{\beta }^{2}|HVH\rangle +{\delta }^{2}|VHH\rangle +{\eta }^{2}|VVV\rangle .$$

Bob and Charlie perform unitary transformation ${\sigma }_{z}^{B}\otimes {\sigma }_{z}^{C}$ on photons *b*_1_ and *c*_1_ of state ${|{\phi }_{11}^{\mathrm{(4)}}\rangle }_{{a}_{1}{b}_{1}{c}_{1}}^{1}$ to get ${|{\phi }_{11}^{\mathrm{(1)}}\rangle }_{{a}_{1}{b}_{1}{c}_{1}}^{1}$.

For the three states ${|{\phi }_{2}\rangle }_{{a}_{1}{b}_{1}{c}_{1}{a}_{2}{b}_{2}{c}_{2}}$, ${|{\phi }_{3}\rangle }_{{a}_{1}{b}_{1}{c}_{1}{a}_{2}{b}_{2}{c}_{2}}$ and ${|{\phi }_{4}\rangle }_{{a}_{1}{b}_{1}{c}_{1}{a}_{2}{b}_{2}{c}_{2}}$, we have the similar results

15 $$\begin{array}{l}{|{\phi }_{11}^{\mathrm{(1)}}\rangle }_{{a}_{1}{b}_{1}{c}_{1}}^{2}=\alpha \beta (|HHV\rangle +|HVH\rangle )+\delta \eta (|VHH\rangle +|VVV\rangle ),\\ {|{\phi }_{11}^{\mathrm{(1)}}\rangle }_{{a}_{1}{b}_{1}{c}_{1}}^{3}=\alpha \delta (|HHV\rangle +|VHH\rangle )+\beta \eta (|HVH\rangle +|VVV\rangle ),\\ {|{\phi }_{11}^{\mathrm{(1)}}\rangle }_{{a}_{1}{b}_{1}{c}_{1}}^{4}=\alpha \eta (|HHV\rangle +|VVV\rangle )+\beta \delta (|HVH\rangle +|VHH\rangle ).\end{array}$$

The four quantum states ${|{\phi }_{11}^{\mathrm{(1)}}\rangle }_{{a}_{1}{b}_{1}{c}_{1}}^{1}$, ${|{\phi }_{11}^{\mathrm{(1)}}\rangle }_{{a}_{1}{b}_{1}{c}_{1}}^{2}$, ${|{\phi }_{11}^{\mathrm{(1)}}\rangle }_{{a}_{1}{b}_{1}{c}_{1}}^{3}$ and ${|{\phi }_{11}^{\mathrm{(1)}}\rangle }_{{a}_{1}{b}_{1}{c}_{1}}^{4}$ are not destroyed by quantum non-demolition detections. They are used as the initial states in the second step of the first round and rewritten as ${|{\phi }_{12}\rangle }_{{a}_{1}{b}_{1}{c}_{1}}^{1}$, ${|{\phi }_{12}\rangle }_{{a}_{1}{b}_{1}{c}_{1}}^{2}$, ${|{\phi }_{12}\rangle }_{{a}_{1}{b}_{1}{c}_{1}}^{3}$ and ${|{\phi }_{12}\rangle }_{{a}_{1}{b}_{1}{c}_{1}}^{4}$.

In the second step, for quantum state

16 $${|{\phi }_{12}\rangle }_{{a}_{1}{b}_{1}{c}_{1}}^{1}=\tfrac{1}{\sqrt{{\alpha }^{4}+{\beta }^{4}+{\delta }^{4}+{\eta }^{4}}}({\alpha }^{2}|HHV\rangle +{\beta }^{2}|HVH\rangle +{\delta }^{2}|VHH\rangle +{\eta }^{2}|VVV\rangle ),$$

photons are all flipped by *HWP*_90°_ and relabeled as *a*_2_, *b*_2_ and *c*_2_. We will get

17 $${|{\phi }_{12}\rangle }_{{a}_{2}{b}_{2}{c}_{2}}^{1}=\tfrac{1}{\sqrt{{\alpha }^{4}+{\beta }^{4}+{\delta }^{4}+{\eta }^{4}}}({\alpha }^{2}|VVH\rangle +{\beta }^{2}|VHV\rangle +{\delta }^{2}|HVV\rangle +{\eta }^{2}|HHH\rangle ).$$

After parity checks and PBSs measurement, we obtain four quantum states

18 $$\begin{array}{l}{|{\phi }_{12}^{\mathrm{(1)}}\rangle }_{{a}_{1}{b}_{1}{c}_{1}}^{1}=\tfrac{1}{{\alpha }^{4}+{\beta }^{4}+{\delta }^{4}+{\eta }^{4}}[{\alpha }^{4}|HHV\rangle +{\beta }^{4}|HVH\rangle +{\delta }^{4}|VHH\rangle +{\eta }^{4}|VVV\rangle ],\\ {|{\phi }_{12}^{\mathrm{(2)}}\rangle }_{{a}_{1}{b}_{1}{c}_{1}}^{1}=\tfrac{1}{{\alpha }^{4}+{\beta }^{4}+{\delta }^{4}+{\eta }^{4}}[{\alpha }^{2}{\beta }^{2}(|HHV\rangle +|HVH\rangle )+{\delta }^{2}{\eta }^{2}(|VHH\rangle +|VVV\rangle )],\\ {|{\phi }_{12}^{\mathrm{(3)}}\rangle }_{{a}_{1}{b}_{1}{c}_{1}}^{1}=\tfrac{1}{{\alpha }^{4}+{\beta }^{4}+{\delta }^{4}+{\eta }^{4}}[{\alpha }^{2}{\delta }^{2}(|HHV\rangle +|VHH\rangle )+{\beta }^{2}{\eta }^{2}(|HVH\rangle +|VVV\rangle )],\\ {|{\phi }_{12}^{\mathrm{(4)}}\rangle }_{{a}_{1}{b}_{1}{c}_{1}}^{1}=\tfrac{1}{{\alpha }^{4}+{\beta }^{4}+{\delta }^{4}+{\eta }^{4}}[{\alpha }^{2}{\eta }^{2}(|HHV\rangle +|VVV\rangle )+{\beta }^{2}{\delta }^{2}(|HVH\rangle +|VHH\rangle )].\end{array}$$

The probabilities of getting quantum states ${|{\phi }_{12}^{\mathrm{(1)}}\rangle }_{{a}_{1}{b}_{1}{c}_{1}}^{1}$, ${|{\phi }_{12}^{\mathrm{(2)}}\rangle }_{{a}_{1}{b}_{1}{c}_{1}}^{1}$, ${|{\phi }_{12}^{\mathrm{(3)}}\rangle }_{{a}_{1}{b}_{1}{c}_{1}}^{1}$ and ${|{\phi }_{12}^{\mathrm{(4)}}\rangle }_{{a}_{1}{b}_{1}{c}_{1}}^{1}$ are

19 $$\begin{array}{l}{p}_{12}^{1}=\frac{{\alpha }^{8}+{\beta }^{8}+{\delta }^{8}+{\eta }^{8}}{{({\alpha }^{4}+{\beta }^{4}+{\delta }^{4}+{\eta }^{4})}^{2}},\\ {p}_{12}^{2}=\frac{\mathrm{2(}{\alpha }^{4}{\beta }^{4}+{\delta }^{4}{\eta }^{4})}{{({\alpha }^{4}+{\beta }^{4}+{\delta }^{4}+{\eta }^{4})}^{2}},\\ {p}_{12}^{3}=\frac{\mathrm{2(}{\alpha }^{4}{\delta }^{4}+{\beta }^{4}{\eta }^{4})}{{({\alpha }^{4}+{\beta }^{4}+{\delta }^{4}+{\eta }^{4})}^{2}},\\ {p}_{12}^{4}=\frac{\mathrm{2(}{\alpha }^{4}{\eta }^{4}+{\beta }^{4}{\delta }^{4})}{{({\alpha }^{4}+{\beta }^{4}+{\delta }^{4}+{\eta }^{4})}^{2}}.\end{array}$$

These are all failed cases, but they can be used as the initial states in the second round.

For quantum state

20 $${|{\phi }_{12}\rangle }_{{a}_{1}{b}_{1}{c}_{1}}^{2}=\tfrac{\alpha \beta }{\sqrt{\mathrm{2(}{\alpha }^{2}{\beta }^{2}+{\delta }^{2}{\eta }^{2})}}(|HHV\rangle +|HVH\rangle )+\tfrac{\delta \eta }{\sqrt{\mathrm{2(}{\alpha }^{2}{\beta }^{2}+{\delta }^{2}{\eta }^{2})}}(|VHH\rangle +|VVV\rangle ),$$

its process of concentration is the same as ${|{\phi }_{12}\rangle }_{{a}_{1}{b}_{1}{c}_{1}}^{1}$ and we can get

21 $${|{\phi }_{12}^{\mathrm{(1)}}\rangle }_{{a}_{1}{b}_{1}{c}_{1}{a}_{2}{b}_{2}{c}_{2}}^{2}=\tfrac{\alpha \beta \delta \eta }{\mathrm{2(}{\alpha }^{2}{\beta }^{2}+{\delta }^{2}{\eta }^{2})}(|HHV\rangle +|HVH\rangle +|VHH\rangle +|VVV\rangle ).$$

This is the maximally GHZ entangled state. The success and failure probabilities of ${|{\phi }_{12}\rangle }_{{a}_{1}{b}_{1}{c}_{1}}^{2}$ are

22 $${p}_{\mathrm{12,}s}^{2}=\frac{\mathrm{2(}\alpha \beta \delta \eta {)}^{2}}{{({\alpha }^{2}{\beta }^{2}+{\delta }^{2}{\eta }^{2})}^{2}},\quad {p}_{\mathrm{12,}f}^{2}=\frac{{\alpha }^{4}{\beta }^{4}+{\delta }^{4}{\eta }^{4}}{{({\alpha }^{2}{\beta }^{2}+{\delta }^{2}{\eta }^{2})}^{2}},$$

where the subscripts *s* and *f* represent the success and failure probabilities respectively.

For quantum states

23 $$\begin{array}{l}{|{\phi }_{12}\rangle }_{{a}_{1}{b}_{1}{c}_{1}}^{3}=\tfrac{\alpha \delta }{\sqrt{\mathrm{2(}{\alpha }^{2}{\delta }^{2}+{\beta }^{2}{\eta }^{2})}}(|HHV\rangle +|VHH\rangle )+\tfrac{\beta \eta }{\sqrt{\mathrm{2(}{\alpha }^{2}{\delta }^{2}+{\beta }^{2}{\eta }^{2})}}(|HVH\rangle +|VVV\rangle ),\\ {|{\phi }_{12}\rangle }_{{a}_{1}{b}_{1}{c}_{1}}^{4}=\tfrac{\alpha \eta }{\sqrt{\mathrm{2(}{\alpha }^{2}{\eta }^{2}+{\beta }^{2}{\delta }^{2})}}(|HHV\rangle +|VVV\rangle )+\tfrac{\beta \delta }{\sqrt{\mathrm{2(}{\alpha }^{2}{\eta }^{2}+{\beta }^{2}{\delta }^{2})}}(|HVH\rangle +|VHH\rangle ),\end{array}$$

the success and failure probabilities of ${|{\phi }_{12}\rangle }_{{a}_{1}{b}_{1}{c}_{1}}^{3}$ and ${|{\phi }_{12}\rangle }_{{a}_{1}{b}_{1}{c}_{1}}^{4}$ are respectively

24 $$\begin{array}{l}{p}_{\mathrm{12,}s}^{3}=\frac{\mathrm{2(}\alpha \beta \delta \eta {)}^{2}}{{({\alpha }^{2}{\delta }^{2}+{\beta }^{2}{\eta }^{2})}^{2}},\quad {p}_{\mathrm{12,}f}^{3}=\frac{{\alpha }^{4}{\delta }^{4}+{\beta }^{4}{\eta }^{4}}{{({\alpha }^{2}{\delta }^{2}+{\beta }^{2}{\eta }^{2})}^{2}},\\ {p}_{\mathrm{12,}s}^{4}=\frac{\mathrm{2(}\alpha \beta \delta \eta {)}^{2}}{{({\alpha }^{2}{\eta }^{2}+{\beta }^{2}{\delta }^{2})}^{2}},\quad {p}_{\mathrm{12,}f}^{4}=\frac{{\alpha }^{4}{\eta }^{4}+{\beta }^{4}{\delta }^{4}}{{({\alpha }^{2}{\eta }^{2}+{\beta }^{2}{\delta }^{2})}^{2}}.\end{array}$$

The total success probability of the first round is

25 $$\begin{array}{l}{P}_{1}={p}_{11}^{2}{p}_{\mathrm{12,}s}^{2}+{p}_{11}^{3}{p}_{\mathrm{12,}s}^{3}+{p}_{11}^{4}{p}_{\mathrm{12,}s}^{4}\\ \quad =\frac{\mathrm{4(}\alpha \beta \delta \eta {)}^{2}}{{\alpha }^{2}{\beta }^{2}+{\delta }^{2}{\eta }^{2}}+\frac{\mathrm{4(}\alpha \beta \delta \eta {)}^{2}}{{\alpha }^{2}{\delta }^{2}+{\beta }^{2}{\eta }^{2}}+\frac{\mathrm{4(}\alpha \beta \delta \eta {)}^{2}}{{\alpha }^{2}{\eta }^{2}+{\beta }^{2}{\delta }^{2}}.\end{array}$$

## Discussion

### Blindness and correctness analysis of the proposed BQC protocol

In the following, we will show that the proposed BQC protocol is secure by analyzing the blindness and correctness.

First, we show the blindness of the proposed BQC protocol.

1. Bob performs one of four Pauli operations randomly chosen by Alice on his photons and the initial states ${|GHZ\rangle }_{{A}_{{t}_{2}}^{\prime }{B}_{j}{C}_{{t}_{1}}^{\prime }}=\frac{1}{2}(|001\rangle +|010\rangle +|100\rangle +|111\rangle )$ are correspondingly changed into one of $\{{|GH{Z}_{1}\rangle }_{{A}_{{t}_{2}}^{\prime }{B}_{j}{C}_{{t}_{1}}^{\prime }}$, ${|GH{Z}_{2}\rangle }_{{A}_{{t}_{2}}^{\prime }{B}_{j}{C}_{{t}_{1}}^{\prime }}$, ${|GH{Z}_{3}\rangle }_{{A}_{{t}_{2}}^{\prime }{B}_{j}{C}_{{t}_{1}}^{\prime }}$, ${|GH{Z}_{4}\rangle }_{{A}_{{t}_{2}}^{\prime }{B}_{j}{C}_{{t}_{1}}^{\prime }}\}$. Whether Bob colludes with Charlie or not, they guess the correct Bell state with the probability of $\tfrac{1}{4}$. When this BQC protocol is repeated *n* times, the probability of obtaining correct quantum states is $\mathop{\mathrm{lim}}\limits_{n\to \infty }\,{(\tfrac{1}{4})}^{n}=0$.
2. Alice randomly chooses the phase *θ*_*i*_ $(\in \,\{0,\frac{\pi }{4},\frac{2\pi }{4},\frac{3\pi }{4},\ldots ,\frac{7\pi }{4}\})$ and disturbs the order of photons *A*_*j*_, *B*_*j*_, *C*_*j*_. Bob and Charlie know nothing about the states $|{\pm }_{{\theta }_{i}}\rangle$ because of the no-signaling principle. After repeating *n* times, the probability of guessing correct *θ*_*i*_ is $\mathop{\mathrm{lim}}\limits_{n\to \infty }\,{(\tfrac{1}{8})}^{n}=0$. In the process of entanglement concentration, Bob and Charlie cannot eavesdropping any useful information by exchanging their results because of difference of orders of three photons.
3. The structures of blind brickwork states and blind topological states are private for servers. Therefore, Bob and Charlie can’t obtain anything about Alice’s private information whether they communicate with each other or not. The blindness of BFK single-server protocol and blind topological single-server protocol are showed in refs 1 and 2 in detail respectively.

Second, the correctness of quantum computation in BFK single-server protocol and blind topological single-server protocol are presented in refs 1 and 2 in detail.

So this BQC protocol is blind and correct.

### Analysis of the success probabilities in iteration

In the above discussion, we have already elaborated the first round of the entanglement concentration with cross-Kerr nonlinearity in detail. QND provides a strong tool for us to perform a quantum nondemolition measurement that does not destroy entanglement of photons, which ensures that each step can be operated independently. Here, we analyse the second round and the *k*-th round of entanglement concentration.

For the three cases ${|{\phi }_{21}\rangle }_{{a}_{1}{b}_{1}{c}_{1}}^{2}$, ${|{\phi }_{21}\rangle }_{{a}_{1}{b}_{1}{c}_{1}}^{3}$ and ${|{\phi }_{21}\rangle }_{{a}_{1}{b}_{1}{c}_{1}}^{4}$, only the first step is needed to concentrate the ideal maximally entangled states ${|GHZ\rangle }_{{A}_{{t}_{2}}^{\prime }{B}_{j}{C}_{{t}_{1}}^{\prime }}$. However, we need to implement two steps for the state ${|{\phi }_{21}\rangle }_{{a}_{1}{b}_{1}{c}_{1}}^{1}$. We consider the three states ${|{\phi }_{21}\rangle }_{{a}_{1}{b}_{1}{c}_{1}}^{2}$, ${|{\phi }_{21}\rangle }_{{a}_{1}{b}_{1}{c}_{1}}^{3}$ and ${|{\phi }_{21}\rangle }_{{a}_{1}{b}_{1}{c}_{1}}^{4}$ first.

In the second round, for the quantum states

26 $${|{\phi }_{21}\rangle }_{{a}_{1}{b}_{1}{c}_{1}}^{2}=\tfrac{{\alpha }^{2}{\beta }^{2}}{\sqrt{\mathrm{2(}{\alpha }^{4}{\beta }^{4}+{\delta }^{4}{\eta }^{4})}}(|HHV\rangle +|HVH\rangle )+\tfrac{{\delta }^{2}{\eta }^{2}}{\sqrt{\mathrm{2(}{\alpha }^{2}{\beta }^{4}+{\delta }^{4}{\eta }^{4})}}(|VHH\rangle +|VVV\rangle ),$$

its analysis is the same as the Eq. (20). The success and failure probabilities are

27 $${p}_{\mathrm{21,}s}^{2}=\frac{\mathrm{2(}\alpha \beta \delta \eta {)}^{4}}{{({\alpha }^{4}{\beta }^{4}+{\delta }^{4}{\eta }^{4})}^{2}},\quad {p}_{\mathrm{21,}f}^{2}=\frac{{\alpha }^{8}{\beta }^{8}+{\delta }^{8}{\eta }^{8}}{{({\alpha }^{4}{\beta }^{4}+{\delta }^{4}{\eta }^{4})}^{2}}.$$

In the *k*-th (*k* > 1) round, the success and failure probabilities are

28 $${p}_{k\mathrm{1,}s}^{2}=\frac{\mathrm{2(}\alpha \beta \delta \eta {)}^{{2}^{k}}}{{({\alpha }^{{2}^{k}}{\beta }^{{2}^{k}}+{\delta }^{{2}^{k}}{\eta }^{{2}^{k}})}^{2}},\quad {p}_{k\mathrm{1,}f}^{2}=\frac{{\alpha }^{{2}^{k+1}}{\beta }^{{2}^{k+1}}+{\delta }^{{2}^{k+1}}{\eta }^{{2}^{k+1}}}{{({\alpha }^{{2}^{k}}{\beta }^{{2}^{k}}+{\delta }^{{2}^{k}}{\eta }^{{2}^{k}})}^{2}}.$$

For the quantum states

$$\begin{array}{l}{|{\phi }_{21}\rangle }_{{a}_{1}{b}_{1}{c}_{1}}^{3}=\tfrac{{\alpha }^{2}{\delta }^{2}}{\sqrt{\mathrm{2(}{\alpha }^{4}{\delta }^{4}+{\beta }^{4}{\eta }^{4})}}(|HHV\rangle +|VHH\rangle )+\tfrac{{\beta }^{2}{\eta }^{2}}{\sqrt{\mathrm{2(}{\alpha }^{4}{\delta }^{4}+{\beta }^{4}{\eta }^{4})}}(|HVH\rangle +|VVV\rangle ),\\ {|{\phi }_{21}\rangle }_{{a}_{1}{b}_{1}{c}_{1}}^{4}=\tfrac{{\alpha }^{2}{\eta }^{2}}{\sqrt{\mathrm{2(}{\alpha }^{4}{\eta }^{4}+{\beta }^{4}{\delta }^{4})}}(|HHV\rangle +|VVV\rangle )+\tfrac{{\beta }^{2}{\delta }^{2}}{\sqrt{\mathrm{2(}{\alpha }^{4}{\eta }^{4}+{\beta }^{4}{\delta }^{4})}}(|HVH\rangle +|VHH\rangle ),\end{array}$$

the analyses of entanglement concentration are the same as the Eq. (23), the success and failure probabilities in the second round and the *k*-th round are

29 $$\begin{array}{ll}{p}_{\mathrm{21,}s}^{3}=\frac{\mathrm{2(}\alpha \beta \delta \eta {)}^{4}}{{({\alpha }^{4}{\delta }^{4}+{\beta }^{4}{\eta }^{4})}^{2}}, & {p}_{k\mathrm{1,}s}^{3}=\frac{\mathrm{2(}\alpha \beta \delta \eta {)}^{{2}^{k}}}{{({\alpha }^{{2}^{k}}{\delta }^{{2}^{k}}+{\beta }^{{2}^{k}}{\eta }^{{2}^{k}})}^{2}},\\ {p}_{\mathrm{21,}f}^{3}=\frac{{\alpha }^{8}{\delta }^{8}+{\beta }^{8}{\eta }^{8}}{{({\alpha }^{4}{\delta }^{4}+{\beta }^{4}{\eta }^{4})}^{2}}, & {p}_{k\mathrm{1,}f}^{3}=\frac{{\alpha }^{{2}^{k+1}}{\delta }^{{2}^{k+1}}+{\beta }^{{2}^{k+1}}{\eta }^{{2}^{k+1}}}{{({\alpha }^{{2}^{k}}{\delta }^{{2}^{k}}+{\beta }^{{2}^{k}}{\eta }^{{2}^{k}})}^{2}},\\ {p}_{\mathrm{21,}s}^{4}=\frac{\mathrm{2(}\alpha \beta \delta \eta {)}^{4}}{{({\alpha }^{4}{\eta }^{4}+{\beta }^{4}{\delta }^{4})}^{2}}, & {p}_{k\mathrm{1,}s}^{4}=\frac{\mathrm{2(}\alpha \beta \delta \eta {)}^{{2}^{k}}}{{({\alpha }^{{2}^{k}}{\eta }^{{2}^{k}}+{\beta }^{{2}^{k}}{\delta }^{{2}^{k}})}^{2}},\\ {p}_{\mathrm{21,}f}^{4}=\frac{{\alpha }^{8}{\eta }^{8}+{\beta }^{8}{\delta }^{8}}{{({\alpha }^{4}{\eta }^{4}+{\beta }^{4}{\delta }^{4})}^{2}}, & {p}_{k\mathrm{1,}f}^{4}=\frac{{\alpha }^{{2}^{k+1}}{\eta }^{{2}^{k+1}}+{\beta }^{{2}^{k+1}}{\delta }^{{2}^{k+1}}}{{({\alpha }^{{2}^{k}}{\eta }^{{2}^{k}}+{\beta }^{{2}^{k}}{\delta }^{{2}^{k}})}^{2}}.\end{array}$$

For the quantum states

30 $${|{\phi }_{kj}\rangle }_{{a}_{1}{b}_{1}{c}_{1}}^{1}={\alpha }_{kj}|HHV\rangle +{\beta }_{kj}|HVH\rangle +{\delta }_{kj}|VHH\rangle +{\eta }_{kj}|VVV\rangle ,$$

(where *j* = 1, 2) we give the relevant normalized coefficients and the probabilities of relevant quantum states. The iterative process is the same as the Eq. (3).

In the first step of the *k*-th round, for the quantum states

31 $${|{\phi }_{k1}\rangle }_{{a}_{1}{b}_{1}{c}_{1}}^{1}={\alpha }_{k1}|HHV\rangle +{\beta }_{k1}|HVH\rangle +{\delta }_{k1}|VHH\rangle +{\eta }_{k1}|VVV\rangle ,$$

where *k* > 1 and the coefficients are

32 $$\begin{array}{l}{\alpha }_{k1}=\frac{{\alpha }^{{2}^{2k-2}}}{\sqrt{{\alpha }^{{2}^{2k-1}}+{\beta }^{{2}^{2k-1}}+{\delta }^{{2}^{2k-1}}+{\eta }^{{2}^{2k-1}}}},\\ {\beta }_{k1}=\frac{{\beta }^{{2}^{2k-2}}}{\sqrt{{\alpha }^{{2}^{2k-1}}+{\beta }^{{2}^{2k-1}}+{\delta }^{{2}^{2k-1}}+{\eta }^{{2}^{2k-1}}}},\\ {\delta }_{k1}=\frac{{\delta }^{{2}^{2k-2}}}{\sqrt{{\alpha }^{{2}^{2k-1}}+{\beta }^{{2}^{2k-1}}+{\delta }^{{2}^{2k-1}}+{\eta }^{{2}^{2k-1}}}},\\ {\eta }_{k1}=\frac{{\eta }^{{2}^{2k-2}}}{\sqrt{{\alpha }^{{2}^{2k-1}}+{\beta }^{{2}^{2k-1}}+{\delta }^{{2}^{2k-1}}+{\eta }^{{2}^{2k-1}}}}.\end{array}$$

In the second step of the *k*-th round, for the quantum states

33 $${|{\phi }_{k2}\rangle }_{{a}_{1}{b}_{1}{c}_{1}}^{1}={\alpha }_{k2}|HHV\rangle +{\beta }_{k2}|HVH\rangle +{\delta }_{k2}|VHH\rangle +{\eta }_{k2}|VVV\rangle ,$$

where the coefficients are

34 $$\begin{array}{l}{\alpha }_{k2}=\frac{{\alpha }^{{2}^{2k-1}}}{\sqrt{{\alpha }^{{2}^{2k}}+{\beta }^{{2}^{2k}}+{\delta }^{{2}^{2k}}+{\eta }^{{2}^{2k}}}},\\ {\beta }_{k2}=\frac{{\beta }^{{2}^{2k-1}}}{\sqrt{{\alpha }^{{2}^{2k}}+{\beta }^{{2}^{2k}}+{\delta }^{2k}+{\eta }^{{2}^{2k}}}},\\ {\delta }_{k2}=\frac{{\delta }^{{2}^{2k-1}}}{\sqrt{{\alpha }^{{2}^{2k}}+{\beta }^{{2}^{2k}}+{\delta }^{{2}^{2k}}+{\eta }^{{2}^{2k}}}},\\ {\eta }_{k2}=\frac{{\eta }^{{2}^{2k-1}}}{\sqrt{{\alpha }^{{2}^{2k}}+{\beta }^{{2}^{2k}}+{\delta }^{{2}^{2k}}+{\eta }^{{2}^{2k}}}}.\end{array}$$

The probabilities of obtaining four quantum states in the first step or the second step of the *k*-th round are

35 $$\begin{array}{c}{p}_{kj}^{1}={\alpha }_{kj}^{4}+{\beta }_{kj}^{4}+{\delta }_{kj}^{4}+{\eta }_{kj}^{4},\\ {p}_{kj}^{2}=2({\alpha }_{kj}^{2}{\beta }_{kj}^{2}+{\delta }_{kj}^{2}{\eta }_{kj}^{2}),\\ {p}_{kj}^{3}=2({\alpha }_{kj}^{2}{\delta }_{kj}^{2}+{\beta }_{kj}^{2}{\eta }_{kj}^{2}),\\ {p}_{kj}^{4}=2({\alpha }_{kj}^{2}{\eta }_{kj}^{2}+{\beta }_{kj}^{2}{\delta }_{kj}^{2}),\end{array}$$

where *j* = 1, 2. The success probability of the *k*th round is

36 $$\begin{array}{rcl}{P}_{k} & = & {p}_{11}^{2}{p}_{\mathrm{12,}f}^{2}{p}_{\mathrm{21,}f}^{2}{p}_{\mathrm{31,}f}^{2}\cdots {p}_{(k-\mathrm{1)1,}f}^{2}{p}_{k\mathrm{1,}s}^{2}\\  &  & +{p}_{11}^{3}{p}_{\mathrm{12,}f}^{3}{p}_{\mathrm{21,}f}^{3}\cdots {p}_{(k-\mathrm{1)1,}f}^{3}{p}_{k\mathrm{1,}s}^{3}\\  &  & +{p}_{11}^{4}{p}_{\mathrm{12,}f}^{4}{p}_{\mathrm{21,}f}^{4}+\cdots {p}_{(k-\mathrm{1)1,}f}^{4}{p}_{k\mathrm{1,}s}^{4}\\  &  & +{p}_{11}^{1}({p}_{12}^{2}{p}_{\mathrm{21,}f}^{2}{p}_{\mathrm{31,}f}^{2}\cdots {p}_{(k-\mathrm{1)1,}f}^{2}{p}_{k\mathrm{1,}s}^{2}\\  &  & +{p}_{12}^{3}{p}_{\mathrm{21,}f}^{3}{p}_{\mathrm{31,}f}^{3}\cdots {p}_{(k-\mathrm{1)1,}f}^{3}{p}_{k\mathrm{1,}f}^{3}\\  &  & +{p}_{12}^{4}{p}_{\mathrm{21,}f}^{4}{p}_{\mathrm{31,}f}^{4}\cdots {p}_{(k-\mathrm{1)1,}f}^{4}{p}_{k\mathrm{1,}f}^{4})\\  &  & +\cdots +{p}_{11}^{1}{p}_{12}^{1}{p}_{21}^{1}{p}_{22}^{1}{p}_{31}^{1}{p}_{32}^{1}\cdots {p}_{(k-\mathrm{1)1}}^{1}{p}_{(k-\mathrm{1)2}}^{1}({p}_{k1}^{2}{p}_{k\mathrm{2,}s}^{2}+{p}_{k1}^{3}{p}_{k\mathrm{2,}s}^{3}+{p}_{k1}^{4}{p}_{k\mathrm{2,}s}^{4}\mathrm{)}.\end{array}$$

**Figure 4:**
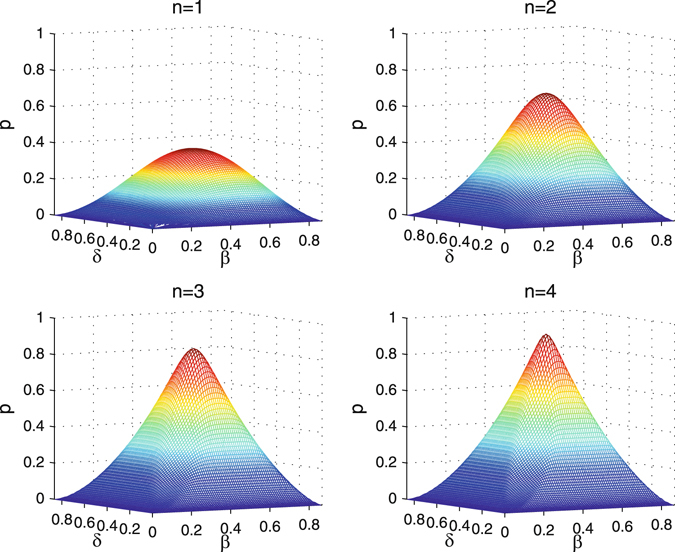
The success probability *P* of getting maximally entangled GHZ state relies on the initial coefficients *β* and *δ*. Here, we let $\alpha =\tfrac{1}{2}$, $\beta \in \{0,\tfrac{\sqrt{3}}{2}\}$, $\delta \in \{0,\sqrt{\tfrac{3}{4}-{\beta }^{2}}\}$, $\eta =\sqrt{\tfrac{3}{4}-{\beta }^{2}-{\delta }^{2}}$. *n* (*n* = 1, 2, 3, 4) represents the number of iterations.

The total probability is ${P}_{total}={\sum }_{k=1}^{n}{P}_{k}$, which depends on the number of iterations and parameters of the initial states. The relationship of the total success probability, parameters and the number of iterations is shown in Fig. 4. It can be seen that the total success probability has kept increasing with the parameters *β* and *δ* in the range of $[\mathrm{0,}\tfrac{\sqrt{3}}{2}]$. When *n* = 4, the success probability has already reached 0.9196. When *n* = 9, the success probability has already reached 0.9975. Therefore, the entanglement concentration is successful in theory.

In this paper, we only consider the ideal CNOT gate^19–23^. In experiment, there exist many nonideal factors such as the double effect of parameter conversion, the imperfect matching of the crystal lattice and phases, and so on. The probabilities of intrinsic error of experimental methods are unavoidable, such as QND measurements and CNOT operations. Thus optimizing the experimental system is a very meaningful research direction. In the BQC protocol, we only give the concrete quantum channel noise model but not universal. So, we will further study entanglement purification of GHZ states.

## Methods

The optical devices are used to complete the entanglement concentration, where the parity check devices are based on cross-Kerr nonlinearity that can construct QND^38, 39, 41^ to improve the successful probability. The cross-Kerr nonlinearity medium is described by the Hamiltonian,

37 $$H=\hslash \chi {a}_{s}^{\dagger }{a}_{s}{a}_{p}^{\dagger }{a}_{p}$$

where ${a}_{s}^{\dagger }$ and ${a}_{p}^{\dagger }$ are the creation operators, *a*_*s*_ and *a*_*p*_ are the annihilation operators, a Fock state |*n*〉 and a coherent state |*α*_*c*_〉 interact. The whole system evolves into

38 $$U(t)|n\rangle |{\alpha }_{c}\rangle ={c}_{0}|0\rangle |{\alpha }_{c}\rangle +{c}_{1}|1\rangle |{\alpha }_{c}{e}^{i\theta }\rangle $$

where $U(t)={e}^{-i\theta {a}_{s}^{+}{a}_{s}{a}_{p}^{+}{a}_{p}}$, *θ* = *χt* is the phase shift and *t* is the interaction time (*c* = 1, 2, 3). *θ* is proportional to the number of photons in the signal state |*α*_*c*_〉. *X*-quadrature measurement can recognize the phase shift of signal states |*α*_*c*_〉. The cross-Kerr nonlinearity can measure the number of photons but do not destroy the photons.

For the parity check device in Fig. 3, we give an example. Two polarization photons are initially prepared with the forms of ${|\tau \rangle }_{{k}_{1}}={\mu }_{0}|H\rangle +{\mu }_{1}|V\rangle$ and ${|\tau \rangle }_{{k}_{2}}={\lambda }_{0}|H\rangle +{\lambda }_{1}|V\rangle$ that interact with a coherent beam |*α*_*c*_〉 (*c* = 1, 2, 3),where real numbers *μ*_0_, *μ*_1_, *λ*_0_ and *λ*_1_ satisfy the normalization condition |*μ*_0_|^2^ + |*μ*_1_|^2^ = 1, |*λ*_0_|^2^ + |*λ*_1_|^2^ = 1, respectively. Then the composite quantum system $|{\Upsilon }_{1}\rangle ={|\tau \rangle }_{{k}_{1}}\otimes {|\tau \rangle }_{{k}_{2}}\otimes |{\alpha }_{c}\rangle$ evolves to

39 $$|{\Upsilon }_{2}\rangle ={\mu }_{0}{\lambda }_{1}|HV\rangle |{\alpha }_{c}{e}^{-2i\theta }\rangle +{\mu }_{1}{\lambda }_{0}|VH\rangle |{\alpha }_{c}{e}^{2i\theta }\rangle +({\mu }_{0}{\lambda }_{0}|HH\rangle +{\mu }_{1}{\lambda }_{1}|VV\rangle )|{\alpha }_{c}\rangle $$

From the Eq. (39), we can pick up a phase shift 0 related with |*HH*〉 and |*VV*〉, and phase shift 2*θ* related with |*HV*〉 and |*VH*〉. One can distinguish |*HH*〉 and |*VV*〉 from |*HV*〉 and |*VH*〉 by different phase shifts, however, the states $|{\alpha }_{1}{e}^{\pm 2i\theta }\rangle$ can not be distinguished by the setup.

## References

[1] Broadbent, A., Fitzsimons, J. & Kashefi, E. Universal blind quantum computation. In *Proceedings of the 50th Annual IEEE Symposium on Foundations of Computer Science* 517–526 (IEEE Computer society, Los Alamitos, USA, 2009).

[2] Morimae T, Fujii K (2012). Blind topological measurement-based quantum computation. Nat. Commun..

[3] Morimae T, Dunjko V, Kashefi E (2015). Ground state blind quantum computation on AKLT state. Quantum Inf. Computat..

[4] Morimae T, Fujii K (2013). Secure entanglement distillation for double-server blind quantum computation. Phys. Rev. Lett..

[5] Li Q, Chan WH, Wu CH, Wen ZH (2014). Triple-server blind quantum computation using entanglement swapping. Phys. Rev. A.

[6] Sheng YB, Zhou L (2015). Deterministic entanglement distillation for secure double-server blind quantum computation. Sci. Rep..

[7] Barz S, Kashefi E, Broadbent A, Fitzsimons JF, Zeilinger A (2012). Demonstration of blind quantum computing. Science.

[8] Darmawan AS, Bartlett SD (2010). Optical spin-1 chain and its use as a quantum-computational wire. Phys. Rev. A.

[9] Hayashi M, Morimae T (2015). Verifiable measurement-only blind quantum computing with stabilizer testing. Phys. Rev. Lett..

[10] Morimae T (2014). Verification for measurement-only blind quantum computing. Phys. Rev. A.

[11] Greganti C, Roehsner MC, Barz S, Morimae T, Walthe P (2016). Demonstration of measurement-only blind quantum computing. New J. Phys..

[12] Takeuchi, Y., Fujii, K., Morimae, T. & Imoto, N. *Practically verifiable blind quantum computation with acceptance rate amplification*. Preprint at arXiv:1607.01568v1 (2016).

[13] Morimae, T. *Measurement*-*only verifiable blind quantum computing with quantum input verification*. Preprint at arXiv:1606.06467v1 (2016).

[14] Takeuchi Y, Fujii K, Ikuta R, Yamamoto T, Imoto N (2016). Blind quantum computation over a collective-noise channel. Phys. Rev. A.

[15] Pérez-Delgado CA, Fitzsimons JF (2015). Iterated gate teleportation and blind quantum computation. Phys. Rev. Lett..

[16] Morimae T (2012). Continuous-variable blind quantum computation. Phys. Rev. Lett..

[17] Sueki T, Koshiba T, Morimae T (2013). Ancilla-driven universal blind quantum computation. Phys. Rev. A.

[18] Morimae T, Fujii K (2013). Blind quantum computation protocol in which Alice only makes measurements. Phys. Rev. A.

[19] Nemoto K, Munro WJ (2004). Nearly Deterministic Linear Optical Controlled-NOT Gate. Phys. Rev. Lett..

[20] Pittman TB, Jacobs BC, Franson JD (2001). Probabilistic quantum logic operations using polarizing beam splitters. Phys. Rev. A.

[21] DeMarco B (2002). Experimental Demonstration of a Controlled-NOT Wave-Packet Gate. Phys. Rev. Lett..

[22] Zhao Z (2005). Experimental Demonstration of a Nondestructive Controlled-NOT Quantum Gate for Two Independent Photon Qubits. Phys. Rev. Lett..

[23] Testolin MJ, Hill CD, Wellard CJ, Hollenberg LCL (2007). Robust controlled-NOT gate in the presence of large fabrication-induced variations of the exchange interaction strength. Phys. Rev. A.

[24] Bennett CH (1996). Purification of Noisy Entanglement and Faithful Teleportation via Noisy Channels. Phys. Rev. Lett..

[25] Pan JW, Simon C, Zeillinger A (2001). Entanglement purification for quantum communication. Nature (London).

[26] Pan JW, Gasparonl S, Ursin R, Weihs G, Zeillinger A (2003). Experimental entanglement purification of arbitrary unknown states. Nature (London).

[27] Sheng YB, Deng FG (2010). One-step deterministic polarization-entanglement purification using spatial entanglement. Phys. Rev. A.

[28] Sheng YB, Deng FG (2010). Deterministic entanglement purification and complete nonlocal Bell-state analysis with hyperentanglement. Phys. Rev. A.

[29] Bennett CH, Bernstein HJ, Popescu S, Schumacher B (1996). Concentrating partial entanglement by local operations. Phys. Rev. A.

[30] Zhao Z, Pan JW, Zhan MS (2001). Practical scheme for entanglement concentration. Phys. Rev. A.

[31] Sheng YB, Deng FG, Zhou HY (2008). Nonlocal entanglement concentration scheme for partially entangled multipartite systems with nonlinear optics. Phys. Rev. A.

[32] Sheng YB, Zhou L, Zhao SM (2012). Efficient two-step entanglement concentration for arbitrary W states. Phys. Rev. A.

[33] Du FF, Li T, Ren BC, Wei HR, Deng FG (2012). Single-photon-assisted entanglement concentration of a multiphoton system in a partially entangled W state with weak cross-Kerr nonlinearity. J. Opt. Soc. Am. B.

[34] Gu B (2012). Single-photon-assisted entanglement concentration of partially entangled multiphoton W states with linear optics. J. Opt. Soc. Am. B.

[35] Wang TJ, Long GL (2013). Entanglement concentration for arbitrary unknown less-entangled three-photon W states with linear optics. J. Opt. Soc. Am. B.

[36] Zhou L, Sheng YB, Zhao SM (2013). Optimal entanglement concentration for three-photon W states with parity check measurement. Chin. Phys. B.

[37] He B, Bergou JA (2008). Entanglement transformation with no classical communication. Phys. Rev. A.

[38] Deng FG (2012). Optimal nonlocal multipartite entanglement concentration based on projection measurements. Phys. Rev. A.

[39] Li XH, Ghose S (2015). Efficient hyperconcentration of nonlocal multipartite entanglement via the cross-Kerr nonlinearity. Opt. Express.

[40] Li XH, Chen X, Zeng Z (2013). Hyperconcentration for entanglement in two degrees of freedom. J. Opt. Soc. Am. B.

[41] Sheng YB, Zhou L, Zhao SM, Zhang B (2012). Efficient single-photon-assisted entanglement concentration for partially entangled photon pairs. Phys. Rev. A.

[42] Zhao Z (2009). Experimental Realization of Entanglement Concentration and a Quantum Repeater. Phys. Rev. Lett..

[43] Sheng YB, Pan J, Guo R, Zhou L, Wang L (2015). Efficient N-particle W state concentration with different parity check gates. Sci. China Phys. Mech..

[44] Du FF, Deng FG (2015). Heralded entanglement concentration for photon systems with linear-optical elements. Sci. China Phys. Mech..

[45] Cao C (2016). Concentrating partially entangled W-class states on nonlocal atoms using low-Q optical cavity and linear optical elements. Sci. China Phys. Mech..

[46] Xia Y, Song J, Song HS (2007). Remote preparation of the N-particle GHZ state using quantum statistics. Opt. Commun..

[47] Bishop LevS (2009). Proposal for generating and detecting multi-qubit GHZ states in circuit QED. New J. Phys..

[48] Bouwmeester D, Pan JW, Daniell M, Weinfurter H, Zeilinger A (1999). Observation of Three-Photon Greenberger-Horne-Zeilinger Entanglement. Phys. Rev. Lett..

[49] Sheng YB, Guo R, Pan J, Zhou L, Wang XF (2015). Two-step measurement of the concurrence for hyperentangled state. Quantum Inf. Process.

